# Pregnancy and childbirth in a patient after multistep surgery and endovascular treatment of cardiovascular disease

**DOI:** 10.5830/CVJA-2015-084

**Published:** 2016

**Authors:** Piotr Buczkowski,, Mateusz Puślecki, Sebastian Stefaniak, Tomasz Urbanowicz, Marek Jemielity, Jerzy Kulesza, Olga Trojnarska

**Affiliations:** Department of Cardiac Surgery and Transplantology, Poznan University of Medical Sciences, Poznan, Poland; Department of Cardiac Surgery and Transplantology, Poznan University of Medical Sciences, Poznan, Poland; Department of Cardiac Surgery and Transplantology, Poznan University of Medical Sciences, Poznan, Poland; Department of Cardiac Surgery and Transplantology, Poznan University of Medical Sciences, Poznan, Poland; Department of Cardiac Surgery and Transplantology, Poznan University of Medical Sciences, Poznan, Poland; Department of Radiology, Poznan University of Medical Sciences, Poznan, Poland; First Department of Cardiology, Poznan University of Medical Sciences, Poznan, Poland

**Keywords:** pregnancy, childbirth, aortic aneurysm, congenital disease, coarctation, hybrid treatment

## Abstract

Nowadays physicians see an increasing population of patients reaching reproductive age after surgery for complex congenital heart defects. Correction of congenital and acquired cardiovascular defects does not exclude experiencing a safe pregnancy. We present the case of a 27-year-old woman, who, after multistep surgery and endovascular treatment of her cardiovascular system, underwent successful pregnancy and uncomplicated childbirth. Recent developments in medicine and interdisciplinary involvement have allowed women with corrected cardiovascular disease the opportunity to become pregnant and experience safe childbirth.

## Abstract

In the past, girls with complex congenital or acquired heart defects often did not reach reproductive age. Recent developments in intensive paediatric cardiac surgery mean that more girls reach the age of maturity. Interdisciplinary involvement has allowed women with corrected cardiovascular defects the opportunity to become pregnant and experience safe childbirth.

## Case report

A 27 year-old woman in good physical condition was admitted to the operating room of the Department of Cardiac Surgery because of a planned pregnancy. When she was six years old, she was operated on for a defect in the interventricular septum. After 10 months, she underwent surgical correction of aortic coarctation using a Dacron patch. During childhood and adolescence, the patient was normotensive and without any cardiac disease.

At the age of 22 years, new symptoms appeared in the form of hoarseness and periodic aphonia, which suggested the presence of a rare postoperative complication, aortic dilatation on the border of the aortic arch and descending aorta. This suspicion was confirmed by imaging with computed angiography, which demonstrated dilatation of the distal aortic arch and descending aorta, starting at a maximum of 65 to 70 mm, with a normaldiameter (19 mm) descending aorta.

She was involved in two-stage hybrid treatment.^1^ Initially, via median sternotomy, anastomosis was performed between the ascending aorta, brachiocephalic trunk and left common carotid artery, using a bifurcated graft (FlowNit Bioseal 12 mm). Due to extensive collateral circulation, the left subclavian artery was not revascularised.

**Fig. 1.**
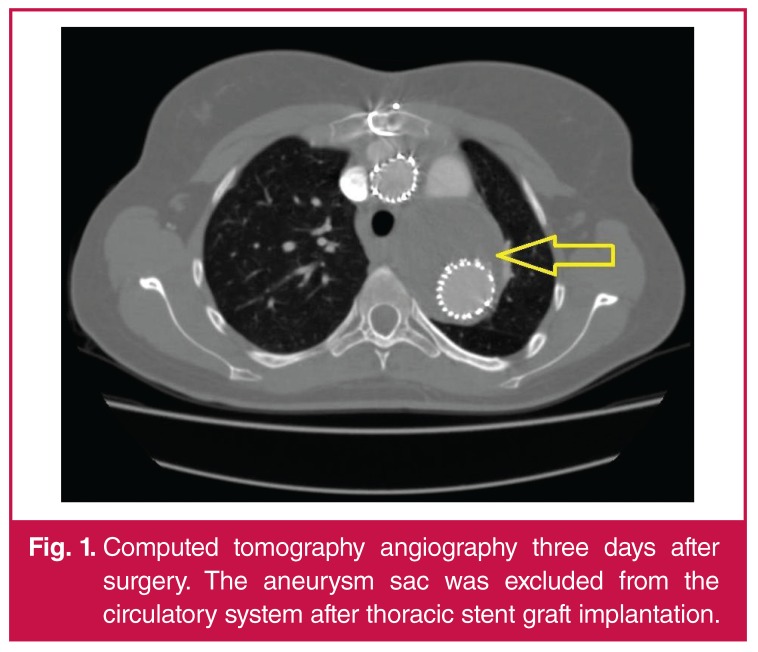
Computed tomography angiography three days after surgery. The aneurysm sac was excluded from the circulatory system after thoracic stent graft implantation.

**Fig. 2.**
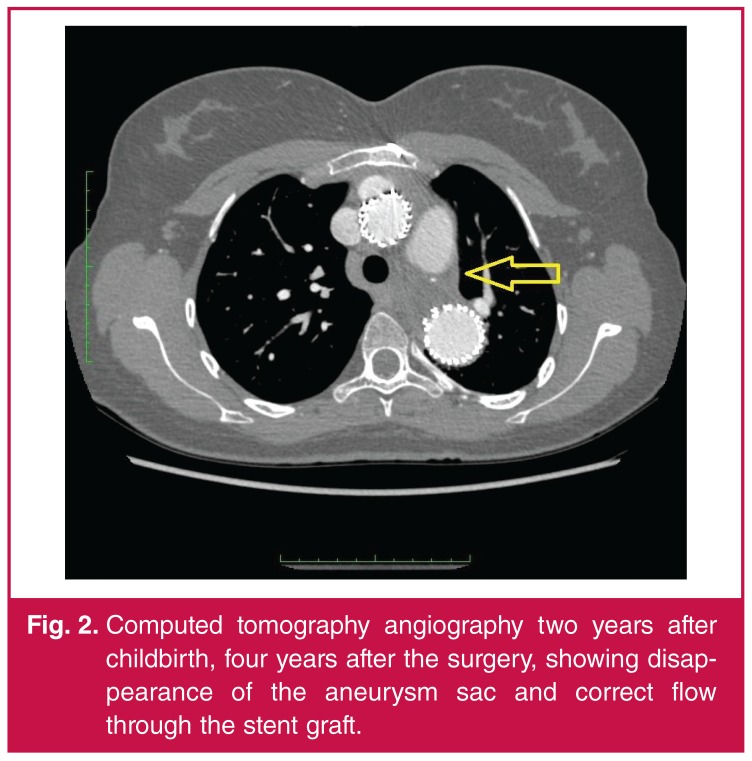
Computed tomography angiography two years after childbirth, four years after the surgery, showing disappearance of the aneurysm sac and correct flow through the stent graft.

**Fig. 3.**
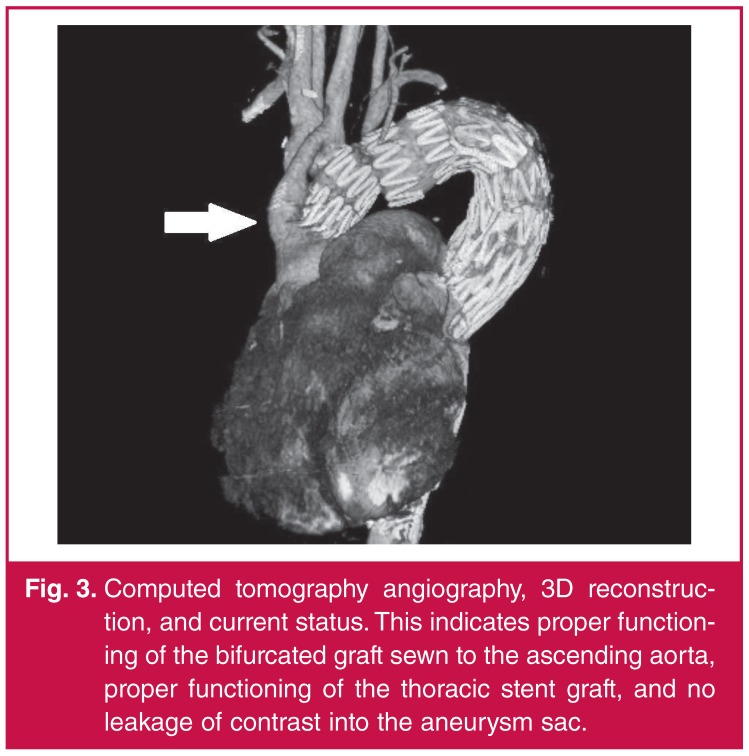
Computed tomography angiography, 3D reconstruction, and current status. This indicates proper functioning of the bifurcated graft sewn to the ascending aorta, proper functioning of the thoracic stent graft, and no leakage of contrast into the aneurysm sac.

After 10 days, the second endovascular step was executed. Two stent grafts (Zenith TX2 TAA 28 mm) were implanted in the aorta using a femoral approach, covering all branches of the arch. The proximal ends of the stent grafts were positioned in the ascending aorta above the previously sewn bifurcated graft (in landing zone 0). The distal end of the graft was placed downstream of the congenital narrowing of the aortic isthmus (Fig. 1, Fig. 2, Fig. 3).

Postoperative hospitalisation and rehabilitation proceeded without complications. A low-dose cardioselective beta-blocker was used as pharmacotherapy. Thereafter, the patient was under the care of Heart Surgery Ambulatory.

Despite the above conditions requiring multi-stage treatment, and the potential complications, the patient consciously decided to become pregnant and was under the constant supervision of an experienced cardiologist who specialises in congenital heart defects in adults. Echocardiography showed normal left ventricular function (left ventricular ejection fraction > 50%), there was no significant gradient of the descending aorta, and the patient was in NYHA functional class I. The cardioselective beta-blocker was discontinued. Pregnancy proceeded without any complications and a decision was made to terminate the pregnancy at 38 weeks’ duration by caesarean section, after an interdisciplinary discussion (cardiologist, obstetrician, cardiac surgeon, neonatologist and patient).

A healthy baby with a birth weight of 2 900 g and 10 points in the APGAR scale score was transferred to the Department of Neonatology. The mother spent the first day in the intensive care unit of Cardiac Surgery. Further hospitalisation proceeded without any complications and she was discharged home on the fourth day. Two years after the birth, control vascular imaging studies confirmed the positive outcome of her previous treatment.

## Discussion

Due to different degrees of potential risk for complications during pregnancy, the European Society of Cardiology (ESC), in a recent guideline, established a four-scale risk score.^2^,^3^ Our patient, because of vascular complications, qualified in the third risk group. A cardiologist, cardiac surgeon and obstetrician specialising in congenital abnormalities, according to the rules in force at that time, took care of the gestation.

The decision was made on the date of termination of pregnancy, taking into account maternal and foetal maturity. Normally, vaginal delivery has a lower risk of complications and the use of epidural anaesthesia is the method of choice. This has been well described in the literature.^4^ However in this case, after interdisciplinary discussion and consultation with the patient, and based on the 2011 ESC guidelines on the management of cardiovascular disease during pregnancy,^2^ some reports in the literature,^5^,^6^ and our experience, we decided to terminate the pregnancy by caesarean section under general anaesthesia.

## Conclusion

The most difficult period for cardiac haemodynamics is the third trimester of pregnancy. Therefore, in the final stage of pregnancy, patients with a cardiovascular history should be treated in specialist departments.
